# Pathology, Molecular Genetics, and Epigenetics of Diffuse Intrinsic Pontine Glioma

**DOI:** 10.3389/fonc.2015.00147

**Published:** 2015-06-30

**Authors:** Pawel Buczkowicz, Cynthia Hawkins

**Affiliations:** ^1^Division of Pathology, The Hospital for Sick Children, Toronto, ON, Canada; ^2^The Arthur and Sonia Labatt Brain Tumour Research Centre, The Hospital for Sick Children, Toronto, ON, Canada; ^3^Department of Laboratory Medicine and Pathobiology, Faculty of Medicine, University of Toronto, Toronto, ON, Canada

**Keywords:** DIPG, glioma, glioblastoma, pediatric, ACVR1, K27M, histone, H3F3A

## Abstract

Diffuse intrinsic pontine glioma (DIPG) is a devastating pediatric brain cancer with no effective therapy. Histological similarity of DIPG to supratentorial high-grade astrocytomas of adults has led to assumptions that these entities possess similar underlying molecular properties and therefore similar therapeutic responses to standard therapies. The failure of all clinical trials in the last 30 years to improve DIPG patient outcome has suggested otherwise. Recent studies employing next-generation sequencing and microarray technologies have provided a breadth of evidence highlighting the unique molecular genetics and epigenetics of this cancer, distinguishing it from both adult and pediatric cerebral high-grade astrocytomas. This review describes the most common molecular genetic and epigenetic signatures of DIPG in the context of molecular subgroups and histopathological diagnosis, including this tumor entity’s unique mutational landscape, copy number alterations, and structural variants, as well as epigenetic changes on the global DNA and histone levels. The increased knowledge of DIPG biology and histopathology has opened doors to new diagnostic and therapeutic avenues.

## Introduction

Diffuse intrinsic pontine gliomas (DIPG), brainstem tumors that diffusely involve the pons, are the most common type of brainstem gliomas (BSG) (1). The mean age of diagnosis for this devastating pediatric neoplasm is 6–7 years (2, 3). The delicate location of these tumors eliminates surgical intervention as a treatment option. Radiation therapy (RT) is the standard of care, although it offers temporary relief of symptoms rather than a real hope of cure (4). Despite 68 clinical trials using various adjuvant chemotherapeutic agents between 1984 and 2014, there has been no improvement in survival compared to radiation alone, and DIPG are currently the number one cause of brain tumor related death in children (5–18). The median survival of DIPG patients is only 10 months post diagnosis and <10% of patients survive 2 years (19, 20). Since the 1980s, the diagnosis of DIPG was based on clinical findings and diagnostic imaging characteristics on computerized tomography (CT) or magnetic resonance imaging (MRI) (4, 21). The lack of surgical and biopsy material has limited most studies of DIPG biology and histology to post-mortem tissue. Although initial investigation into the safety of incorporating biopsy for BSG showed no surgical mortality and low surgical morbidity (22), the advent of CT and MRI allowed for accurate non-invasive localization of BSG (23). It was noted that patients with DIPG represented the majority of deaths in children with BSG and biopsies, which did not stratify patients into different treatment groups, were abandoned (23). Recently, the role for biopsy in DIPG diagnosis has been substantially strengthened due to several important discoveries related to the biology and histopathology of this universally fatal tumor (3, 24, 25) and successful use of stereotactic biopsy by several centers with limited morbidity (26–29). Biologically, DIPG is a unique tumor entity which possesses properties that are antithetical when compared to other brain tumors, such as pediatric and adult supratentorial high-grade astrocytomas (HGA), which they most often resemble histologically. This review highlights the unique pathology, genetics, and epigenetics of DIPG.

## From Candidate Gene Approaches to Next-Generation Sequencing

Early molecular profiling of pediatric gliomas was limited to candidate gene approaches (30–33) focusing on mutational and copy number profiling of genes known to be frequently implicated in adult HGA, namely *EGFR*, *TP53*, *IDH1/2*, *CDKN2A*, *MGMT*, and *PTEN* (34–36). All early studies of DIPG were performed on small cohorts or case studies, which limited their usefulness in defining the biology of this deadly pediatric cancer. However, conclusions from these studies still highlighted some differences between DIPG and adult HGA. Unfortunately, these candidate gene approaches were limited to what was known about adult HGA and future studies using whole-genome profiling approaches would be required to discover more robust differences between these entities. Whole genome profiling technologies such as array CGH and SNP-genotyping allowed for the first genome-wide copy number analysis of cancers. Although giving a low resolution perspective at first, newer iterations allowed for greater and greater resolution and began to highlight the unique molecular profiles of DIPG when compared to pediatric and adult supratentorial astrocytomas. Copy number signatures at the whole chromosome arm level revealed differences between DIPG and adult HGA (37). Gains of chromosome 1q and losses of 11p, 13q, and 14q were frequently observed in DIPG and less so in supratentorial HGA (37). Subsequent copy number studies, with larger DIPG patient tumor cohorts, revealed frequently altered genes (Table 1) including *PDGFRA*, *TP53, PARP1, PVT-1/MYC*, *RB1*, and *PTEN* (3, 38, 39). Most recently, next-generation sequencing approaches, including whole-genome sequencing (WGS), whole-exome sequencing (WES), and RNA-sequencing integrated with histopathology, copy number, gene expression, and methylation profiling, and other molecular techniques have re-defined what we know about DIPG genetics and epigenetics.

**Table 1:** **Frequencies of most common mutations and copy number alterations in diffuse intrinsic pontine glioma from the literature**.

Gene	Alteration type	Frequency in DIPG (%)	Reference
ACVR1	Mutation	20–32	(40–42)
ATRX	Mutation	9–13	(3, 24)
BRAF	Mutation	0	(40–43)
CDK6	Copy number gain	3–4	(3, 37, 44)
CDKN2A/B	Focal deletion	3–4	(37–39)
EGFR	Mutation	0–2	(40–43, 45)
	Amplification	0–2	
FGFR	Fusion	0	(40–43)
H3F3A	Mutation	58–65	(3, 40–43, 46)
HIST1H3B/C	Mutation	12–19	(40–43, 46)
IDH1/2	Mutation	0	(3, 40–43, 46)
MYC/PVT-1	Copy number gain	14	(3, 40)
MYCN	Copy number gain	7	(3, 40)
NF1	Mutation	0–3	(40–43)
	Focal deletion	7	
PDGFRA	Mutation	5–9	(3, 37, 38, 40, 47, 48)
	Copy number gain	28–36	
PIK3CA	Mutation	12–23	(2, 27)
PPM1D	Mutation	10–12	(42, 46)
TP53	Mutation	42–71	(2, 3, 32, 40, 49)
	Heterozygous deletion	35–64	
PTEN	Mutation	0–3	
	Focal deletion	14	(38–43)
		
RB1	Mutation	0	(3, 38–43)
	Focal deletion	16	

## Mutational Landscape

**Figure 1:**
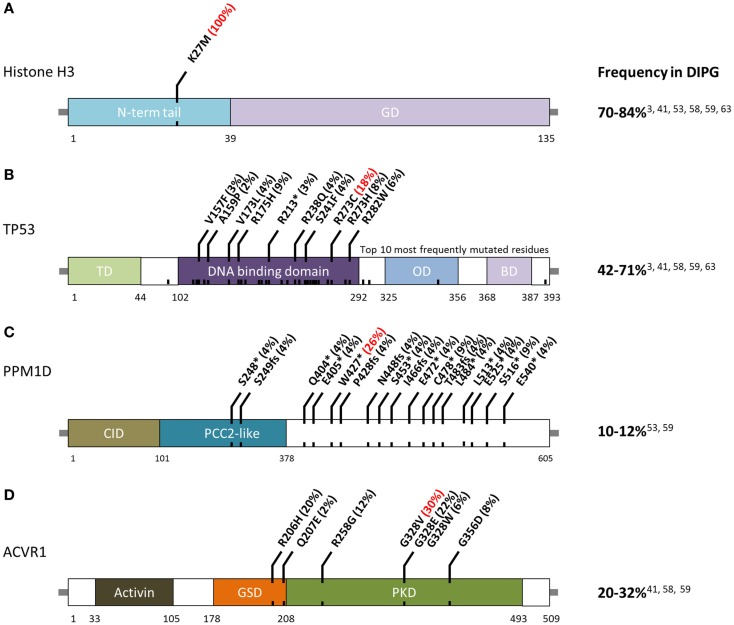
**Schematic of residue alterations in (A) histone H3, (B) TP53, (C) PPM1D, and (D) ACVR1 as a result of mutations most frequently identified in DIPG**. GD, globular domain; TD, transactivation domain; OD, oligomerization domain; BD, basic domain; CID, CHEK1 interacting domain; GSD, GS domain; PKD, protein Kinase domain. Red indicates most frequently altered amino acid.

The first major breakthrough in defining the DIPG mutational landscape came in 2012, when studies on pediatric brain tumors using whole-genome and WES reported that 70–84% of DIPG possess mutations in histone H3, and that these mutations were predictive of outcome (3, 24, 25). These recurrent mutations, in *H3F3A* or *HIST1H3B/C/I* (Figure 1A), result in a p. Lys27Met (K27M) substitution. A subset of other midline astrocytomas, such as those arising in the thalamus, have also been found to harbor K27M histone H3 mutations although at a lower frequency (50). Conversely, pediatric supratentorial HGA rarely possess these mutations, and more frequently have p. Gly34Arg (G34R) or p. Gly34Val (G34V) substitution in histone H3.3. The G34R/V-H3.3 mutations occur in 10–19% of supratentorial HGA cases (24) and are never found in DIPG (3). Furthermore, among supratentorial GBM, these G34R/V-H3.3 mutations are predominantly found in older children and young adults (3). Mutations affecting these two histone residues are extremely rare in adult HGA.

Histones are proteins that form octomeric complexes known as nucleosomes around which DNA wraps and condenses into chromatin. The majority of histone mutations in DIPG, 65%, effect histone variant H3.3 (3, 24, 25). Although this histone is coded in two genes, *H3F3A* and *H3F3B*, K27M mutations are only found in the former. K27M-H3.1 mutations are found in 12–19% of DIPG and are mutually exclusive from K27M-H3.3 mutations (24, 40). Histone H3.1 is coded by a cluster of 10 genes on chromosome 6 known as the *HIST1* cluster (44). Currently, K27M-H3.1 mutations have been detected in *HIST1H3B*, *HIST1H3C*, and *HISTIH3I*.

Histones play a key role in the state of chromatin; however, they themselves do not determine whether chromatin will be in a conformation more permissive to gene expression (euchromatin) or in a conformation less permissive to gene expression (heterochromatin). Several histone marks can be laid on specific residues across the histone tail, including lysine 27, which can be post-translationally modified by either acetylation or mono-, di-, or tri- methylation (51–53). Various cellular machineries are implicated in the reading, writing, and copying of these epigenetic histone marks, including histone acetyltransferases (HATs), histone deacetylases (HDACs), histone methyltransferases (HMTs), and histone demethylases (54). The methionine substitution at K27 in DIPG cannot be modified and several *in vitro* and *in vivo* studies have documented a global decrease in H3K27me3 in the presence of mutant histone (40, 55–58). This global decrease is attributed to polycomb repressive complex 2 (PRC2) inhibition (59); however, other molecular consequences of this mutation, including its effect on histone marks, global DNA methylation, and gene expression, are still under investigation. Histone H3 mutations are undoubtedly important in DIPG tumorigenesis and/or maintenance but in a majority of cases are found to be partnered with other mutations and copy number alterations (CNAs) (3, 40), suggesting that they are not the sole drivers of DIPG tumorigenesis.

*TP53* mutations and CNAs occur in 42–71% of DIPG (Figure 1B) (3, 40). Hemizygous deletions are often associated with a mutation and strong protein expression by immunohistochemistry (32, 49). Interestingly, DIPG with low-grade astrocytoma histology (grade II), even those harboring H3-mutations, are not mutated for *TP53* (2). Among DIPG with high-grade histology (grade III and IV), both H3-mutant and wild-type DIPG often have mutated *TP53* (2). However, a recent exome sequencing study identified *PPM1D* mutations (Figure 1C) in a subset of H3-mutant but *TP53* wild-type DIPG (46). These mutations account of ~50% of *TP53* wild-type grade II DIPG. *PPM1D* gene codes for WIP1 (wild-type p53-induced protein phosphatase 1D) and has been implicated as an oncogene in other cancers (60–62). Mutations of *PPM1D* have been shown to be functionally equivalent to those of *TP53* (46, 63). Taken together, alterations of the *TP53* signaling pathway in DIPG are only slightly less common than histone H3 mutations.

The third most commonly mutated gene in DIPG codes for the activin A receptor, type I (*ACVR1*), a member of the bone morphogenic protein (BMP) signaling pathway. Approximately, 20–32% of DIPG harbor mutations in *ACVR1* which significantly overlap with K27M mutations in histone H3.1 (40–42). Previously only reported in a congenital autosomal dominant disease of the connective tissue called fibrodysplasia ossificans progressiva (FOP), *ACVR1* mutations result in ligand-independent constitutive activation of the BMP signaling pathway (64–66). Although seven different *ACVR1* mutations have been reported in DIPG, the most common alteration in this tumor type, p.Gly328Val, has not been reported in FOP patients. Several other residues of *ACVR1* are frequently mutated in DIPG (Figure 1D). These mutations have been shown to increase levels of phosphorylated SMAD1/5 (40–43) as well as increased gene expression of downstream BMP signaling targets *ID1* and *ID2* (40). Research into the cooperation between *ACVR1* and histone H3.1 mutations, and their effects on tumorigenesis are still ongoing.

## Amplification and Mutations of Receptor Tyrosine Kinases

Receptor tyrosine kinases (RTKs) are cell surface receptors which are often dysregulated in cancers. The RTK/RAS/PI-3K signaling pathway is the most commonly dysregulated signaling pathway in adult GBM, with 90% of tumors exhibiting CNAs or mutations in pathway members (34). Mutations and CNAs in the platelet-derived growth factor receptor alpha (*PDGFRA*) have been implicated in both adult and pediatric HGA. Several array based studies of DIPG revealed amplification of *PDGFRA* in 28–36% of patient tumors (37, 38). The frequency of these amplifications in pediatric and adult supratentorial HGA was reported in the range of 7–14%, and was preferentially identified in the Proneural subtype of adult GBM (36). In DIPG, *PDGFRA* amplifications are exclusively found in patients with K27M-H3 mutations and across all astrocytic histologies (grade II-IV) (2, 40). Oncogenic mutations of *PDGFRA* have also been reported in 5–9% of DIPG (47, 48). Epidermal growth factor receptor (*EGFR*) copy number gains and mutations are among the most frequent alterations in adult HGA, occurring in 60–85% of GBM (34, 35). In adults, EGFR protein expression correlates with amplifications and EGFRvIII mutations; however in DIPG, *EGFR* CNAs and mutations are extremely rare, found in 0–2% of patient tumors (45), and do not correlate with immunopositivity (67, 68). The RTK/RAS/PI-3K signaling pathway is further dysregulated in DIPG through hemizygous deletions of *PTEN* (40), as well as mutations of *PIK3CA* and *PIK3R1* (2, 27). Although this pathway has often been targeted in clinical trials, the majority of the CNAs and mutations in the RTK/RAS/PI-3K pathway are clonal events, as determined by fluorescence *in situ* hybridization (FISH) for *PDGFRA* amplifications (37) and mutation allele frequency of *PIK3CA*, suggesting these events likely arise later in tumor development.

## DNA Damage Repair (PARP1, MGMT, MPG)

Radiotherapy is the standard of care for DIPG and adjuvant chemotherapeutics have been shown to be ineffective. Perturbations in DNA damage repair pathways in DIPG were first identified in 2010 by Zarghooni et al. Loss of heterozygosity (LOH) was identified in many genes involved in nucleotide excision repair, non-homologous end-joining (NHEJ), homologous recombination (HR), base excision repair (BER), and mismatch repair (MMR) by analysis of both SNP arrays and microsatellite markers (37). Poly (ADP-ribose) polymerase (PARP1), a protein essential for repair of single strand DNA breaks induced by alkylating agents, as well as repair of ionizing radiation induced double strand breaks by HR and NHEJ were found to be gained and/or overexpressed in 54% of DIPG, highlighting it as a potential therapeutic target (37). Furthermore, pathway analysis revealed a subset of patients with LOH or deletions in members of the *BRCA* DNA damage response pathway, including *BRCA1* and *BRCA2*. Defects in either of these two genes have been implicated in promoting sensitivity to single strand DNA repair via PARP inhibition.

Temozolomide (TMZ) is a frontline DNA alkylating agent most often used in treatment of adult GBM. TMZ causes DNA damage by alkylating O^6^-guanine, N^7^-guanine, and N^3^-adenine residues. In adults, it was identified that a subgroup of patients with *MGMT* (O6-methylguanine DNA methyltransferase) promoter methylation had improved overall survival when treated with radiotherapy and concomitant TMZ compared to patients without *MGMT* promoter methylation (69). MGMT repairs TMZ alkylated O6-guanine nucleotides. However, the universal lack of response to TMZ in DIPG patients could not be attributed to this resistance mechanism, as DIPG have not been found to express MGMT (37). It has been recently described that TMZ resistance in the pediatric population can be attributed to an ATM-dependent regulation of 3-methylpurine-DNA glycosylase (MPG), an enzyme responsible for repair of alkylated N^7^ guanine and N^3^ adenine residues (70). Further investigation into using radio-sensitizing and chemo-sensitizing agents in DIPG therapy is warranted.

## Isocitrate Dehydrogenase, *ATRX*, and Telomeres

Mutations in the mitochondrial enzyme gene, isocitrate dehydrogenase 1 (*IDH1*), whose gene product catalyzes the oxidative decarboxylation of isocitrate to α-keto glutarate, are found in approximately 70–80% of adult low-grade astrocytomas (LGA) and anaplastic astrocytomas (AA), as well as secondary glioblastoma (GBM) (71). Furthermore, in adult gliomas, *IDH1* mutations were found to be associated with *ATRX* mutations and alternative lengthening of telomeres (ALT) (72). Isocitrate dehydrogenase 2 (*IDH2*) mutations are also found in a subset of adult HGA but to date, no mutations in *IDH1* or *IDH2* have been detected in DIPG.

Mutations of chromatin remodeling genes are less common in DIPG than in supratentorial HGA (3, 24). *ATRX*, which codes for the α-thalassemia/mental retardation syndrome X-linked gene, was found to be mutated in a subset of DIPG (~9%) but in contrast to pediatric supratentorial HGA, had no clear overlap with histone H3 mutations (3). Pediatric supratentorial HGA showed high overlap with *ATRX* or *DAXX* mutations (15–25%) and G34R/V-H3.3 alterations, as well as mutual exclusivity of *IDH1/2* mutations (24, 73). Irrespective of tumor location, in the pediatric population *ATRX* mutations significantly overlap with *TP53* mutations and are predominantly found in older children (3). *ATRX* and *DAXX* (death-domain associated protein) are genes encoding subunits of a chromatin remodeling complex required for histone H3.3 incorporation at telomeric regions. Telomeres are repetitive regions of DNA found on the ends of chromosomes and shorten during every cell division due to incomplete DNA replication (74). Over many cell divisions, telomeres may reach a critically short length, which results in cellular senescence (75). This fate can be avoided by expressing telomerase, an enzyme that can extend telomeres. Although telomerase is not expressed in most mature, terminally differentiated cells (76, 77), its expression and activity has been implicated in several brain cancers as a poor prognostic marker and potential therapeutic target (78–80). Extension of telomeres in certain cancers can be attained by a telomerase independent mechanism known as alternative lengthening of telomeres (ALT). Twenty percent of DIPG test positive for ALT by either TRF (telomere restriction fragment) assay or C-circles assay and ALT positive DIPG are exclusive carriers of the K27M-H3.3 mutation (40). ALT phenotype is also associated with an older age of diagnosis in DIPG and to date has not been detected in patients with low-grade astrocytoma histology (2, 40).

## DIPG Histopathology

**Figure 2:**
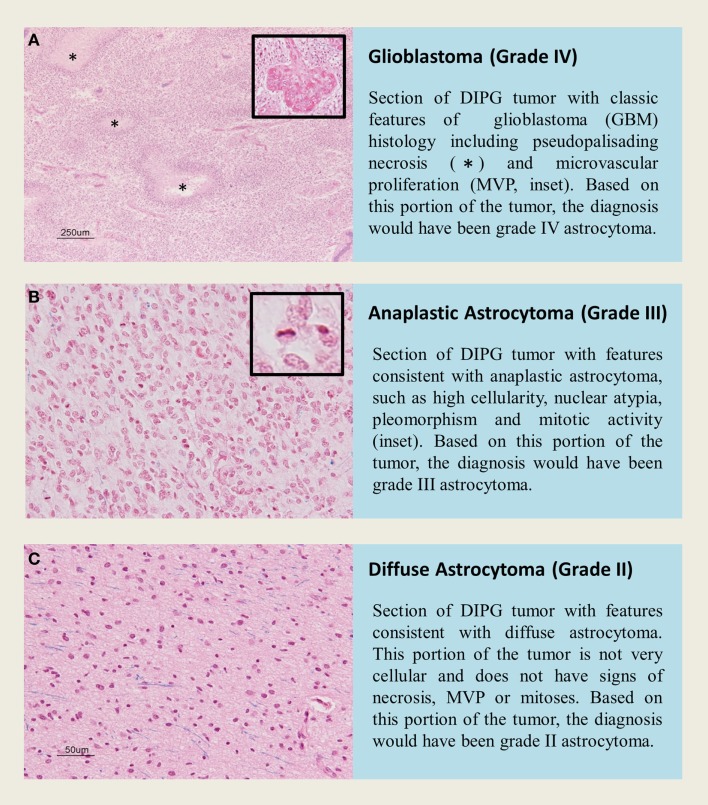
**H&E staining of tissue sections from an autopsy of a K27M-H3.1 mutant diffuse intrinsic pontine glioma patient highlights vast regional differences that histologically resemble (A,B) high grade astrocytoma (WHO grade III–IV) or (C) low grade astrocytoma (WHO grade II)**. Inadvertently targeting these regions on biopsy could lead to misdiagnosis. Histone H3 mutation predicts overall survival better than histologic grade for DIPG. Mutational testing at the time of stereotactic biopsy should be implemented into clinical practice for DIPG patients.

Diffuse intrinsic pontine glioma is a heterogeneous disease, and represents a varied histological spectrum. These tumors are very diffuse and often involve adjacent brain structures beyond the pons. Several studies report leptomeningeal dissemination and subventricular spread as a common occurrence seen in as many as one-third of DIPG, with tumor cells found as far rostrally as the frontal lobe (2, 81, 82). A review by Jansen et al. reported World Health Organization (WHO) central nervous system tumor classification on 108 biopsies from 13 studies, including 37 AA (WHO grade III), 27 GBM(WHO grade IV), 22 LGA (WHO grade II), 3 anaplastic oligoastrocytomas (WHO grade III), and 19 tumors with “not further specified” or undefined characterization (9). Data from this biopsy series would suggest that WHO grade III AA are the most common histological entity in brainstem glioma; however, autopsy based histopathological studies report WHO grade IV GBM to be the most common histology, potentially highlighting the caveat of limited tissue sampling during biopsy or anaplastic progress and/or treatment effect seen in late stage disease at autopsy. Autopsy based studies revealed that GBM histology was most common in DIPG. Of the 33 pediatric patients examined by Yoshimura et al., 29 were reported to be GBM and 4 with anaplastic astrocytoma histology (83). A larger autopsy based study of DIPG histology reported 42 GBM, 18 anaplastic astrocytoma, 8 low-grade astrocytoma, and 2 with features of primitive neuroectodermal tumor (PNET, WHO grade IV) (13). Previous investigations into DIPG histology have also observed this rare PNET histology in the brainstem (84). Importantly, regional differences within one DIPG specimen can bias biopsy based diagnoses. Autopsy studies allow extensive tissue sampling and have highlighted intratumoral histopathologic heterogeneity (2). Areas within or around a grade IV astrocytoma may present with features of grade II or grade III histology that could be inadvertently targeted at biopsy (Figure 2).

Perhaps most importantly, DIPG histology is not a predictor of survival. Patients with low-grade histology do just as poorly as patients with high-grade histology. On multivariate Cox regression analysis, only histone H3 mutation is a predictor of worse overall survival, irrespective of histological grade, age of diagnosis, and sex (2, 3). Furthermore, DIPG patients, which at autopsy had grade II histology, but were mutated for histone H3, had clinical outcomes similar to what would be expected of high-grade tumors (2, 3). This important finding suggests brainstem glioma require their own WHO grading scheme, which needs to incorporate H3-mutation testing at biopsy.

## Molecular Subgroups of DIPG

**Figure 3:**
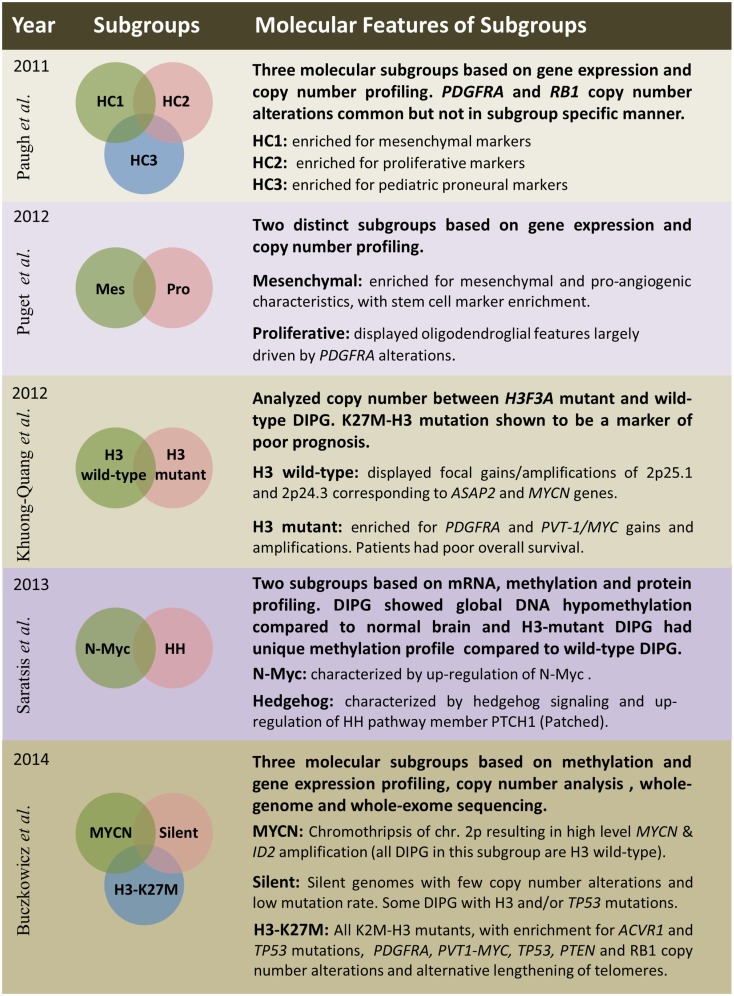
**Summary of five publications between 2011 and 2014 outlining clinical and molecular features of diffuse intrinsic pontine gliomas based on various platforms and sample sizes;** Paugh et al. (38), three subgroups – *n* = 27; Puget et al. (48), two subgroups – *n* = 23; Khuong-Quang et al. (3), two subgroups – *n* = 42; Saratsis et al. (85), two subgroups – *n* = 14; Buczkowicz et al. (40), three subgroups – *n* = 48.

Several studies have embarked on molecular subgrouping of DIPG based on various molecular signatures, including gene expression profiling, copy number analysis, proteomics, mutational profiles, and methylation profiling (Figure 3). A study by Paugh et al. in 2011 profiled 27 DIPG by gene expression arrays and classified them into three subgroups based on hierarchical clustering and compared the subgroup specific enrichment scores to previously identified subgroups of adult GBM, highlighting significant enrichment in either mesenchymal, proliferative, or pronerual markers (38). These data were also compared to genomic copy number abnormalities determined by single-nucleotide polymorphism arrays which highlighted frequent focal amplifications of *PDGFRA* and *RB1*. However, these CNAs were not restricted to any of the three identified subgroups. A subsequent study of gene expression profiles from 23 DIPG matched with copy number data from array CGH identified two subgroups using K-means clustering (48). These two subgroups were enriched for mesenchymal and proneural markers, respectively, with the proneural subgroup displaying oligodendroglial features and alterations of *PDGFRA* (48). With the discovery of histone mutations in ~80% of DIPG in 2012 and their clinical relevance in patient survival, supervised analysis of CNAs in wild-type and histone mutant subgroups by single-nucleotide polymorphism arrays highlighted *PDGFRA* and *PVT-1/MYC* gains and amplifications to be present at high frequency in K27M-H3 mutant DIPG, whereas wild-type DIPG were enriched for *MYCN* amplification as determined by GISTIC2.0 analysis (3). A study from 2013 by Saratsis et al. utilized mRNA and methylation profiles with protein profiling of 14 DIPG specimens. This study also reported two subgroups, which were characterized by upregulation of *N*-Myc or Hedgehog signaling through mRNA expression and DNA hypomethylation (85).

The most comprehensive subgrouping of DIPG integrated CpG island methylation with WGS and WES, gene expression, and copy number profiling to find that DIPG are three molecularly distinct subgroups; MYCN, Silent, and H3-K27M (40). The MYCN subgroup did not contain any DIPG with histone mutations, but was characterized by high-level amplifications of *MYCN* and *ID2* genes caused by chromothripsis on chromosome 2p. Furthermore, these *MYCN* amplified DIPG did not contain *PVT-1/MYC*, *PDGFRA*, or *RB1* CNAs, which were previously described to be common in these brainstem neoplasms (40). The Silent subgroup did not contain many CNAs or mutations. In fact, this subgroup was characterized by stable genomes when compared to both MYCN and H3-K27M subgroup DIPG. Some DIPG in this subgroup contained histone H3, *TP53*, and *ACVR1* mutations; however, they were found at a lower frequency. All patients within the largest subgroup, H3-K27M, had histone H3-mutations. This subgroup was further characterized by hypomethylated genomes and also enriched in *ACVR1* mutations (which significantly co-occurred with K27M-H3.1 mutations), *TP53/PPM1D* mutations/homozygous deletions, alternative lengthening of telomeres, and CNAs of *PVT-1/MYC*, *PTEN*, *PDGFRA*, and *RB1* (40).

## Conclusion

In recent years, the combined increase in knowledge of DIPG histology, genetics, and epigenetics has been substantial. Mutations in previously undiscovered oncogenes, histone H3, and *ACVR1*, molecular subtypes of DIPG, and the rediscovered role for biopsy for this tumor entity are redefining what is clinically possible in treating these patients. The major challenges ahead are developing molecularly accurate pre-clinical models for testing of new therapeutics and integrating new genetic/epigenetic and histopathological knowledge for DIPG patient diagnosis, treatment, and future clinical trial design. In the near future, DIPG patients will benefit from mutational testing and molecular subgrouping at biopsy, which will provide more accurate prognosis and actionable tumor-specific genomic targets for these affected children.

## Conflict of Interest Statement

The authors declare that the research was conducted in the absence of any commercial or financial relationships that could be construed as a potential conflict of interest. The Guest Associate Editor David D. Eisenstat declares that, despite having collaborated with author Cynthia Hawkins, the review process was handled objectively and no conflict of interest exists.

## References

[1] Ramos A Hilario A Lagares A Salvador E Perez-Nunez A Sepulveda J. Brainstem gliomas. Semin Ultrasound CT MR (2013) 34(2):104–12. 10.1053/j.sult.2013.01.001 23522775

[2] Buczkowicz P Bartels U Bouffet E Becher O Hawkins C. Histopathological spectrum of paediatric diffuse intrinsic pontine glioma: diagnostic and therapeutic implications. Acta Neuropathol (2014) 128(4):573–81. 10.1007/s00401-014-1319-6 25047029 PMC4159563

[3] Khuong-Quang DA Buczkowicz P Rakopoulos P Liu XY Fontebasso AM Bouffet E K27M mutation in histone H3.3 defines clinically and biologically distinct subgroups of pediatric diffuse intrinsic pontine gliomas. Acta Neuropathol (2012) 124(3):439–47. 10.1007/s00401-012-0998-0 22661320 PMC3422615

[4] Robison NJ Kieran MW. Diffuse intrinsic pontine glioma: a reassessment. J Neurooncol (2014) 119(1):7–15. 10.1007/s11060-014-1448-8 24792486

[5] Bailey S Howman A Wheatley K Wherton D Boota N Pizer B Diffuse intrinsic pontine glioma treated with prolonged temozolomide and radiotherapy – results of a United Kingdom phase II trial (CNS 2007 04). Eur J Cancer (2013) 49(18):3856–62. 10.1016/j.ejca.2013.08.006 24011536 PMC3853623

[6] Bartels U Wolff J Gore L Dunkel I Gilheeney S Allen J Phase 2 study of safety and efficacy of nimotuzumab in pediatric patients with progressive diffuse intrinsic pontine glioma. Neuro Oncol (2014) 16(11):1554–9. 10.1093/neuonc/nou091 24847085 PMC4201068

[7] Broniscer A Baker SD Wetmore C Pai Panandiker AS Huang J Davidoff AM Phase I trial, pharmacokinetics, and pharmacodynamics of vandetanib and dasatinib in children with newly diagnosed diffuse intrinsic pontine glioma. Clin Cancer Res (2013) 19(11):3050–8. 10.1158/1078-0432.CCR-13-0306 23536435 PMC3685168

[8] Cohen KJ Gibbs IC Fisher PG Hayashi RJ Macy ME Gore L. A phase I trial of arsenic trioxide chemoradiotherapy for infiltrating astrocytomas of childhood. Neuro Oncol (2013) 15(6):783–7. 10.1093/neuonc/not021 23460318 PMC3661102

[9] Jansen MH van Vuurden DG Vandertop WP Kaspers GJ. Diffuse intrinsic pontine gliomas: a systematic update on clinical trials and biology. Cancer Treat Rev (2012) 38(1):27–35. 10.1016/j.ctrv.2011.06.007 21764221

[10] Janssens GO Jansen MH Lauwers SJ Nowak PJ Oldenburger FR Bouffet E Hypofractionation vs conventional radiation therapy for newly diagnosed diffuse intrinsic pontine glioma: a matched-cohort analysis. Int J Radiat Oncol Biol Phys (2013) 85(2):315–20. 10.1016/j.ijrobp.2012.04.006 22682807

[11] Muller K Schlamann A Guckenberger M Warmuth-Metz M Gluck A Pietschmann S Craniospinal irradiation with concurrent temozolomide for primary metastatic pediatric high-grade or diffuse intrinsic pontine gliomas. A first report from the GPOH-HIT-HGG Study Group. Strahlenther Onkol (2014) 190(4):377–81. 10.1007/s00066-013-0513-0 24638239

[12] Okada K Yamasaki K Tanaka C Fujisaki H Osugi Y Hara J. Phase I study of bevacizumab plus irinotecan in pediatric patients with recurrent/refractory solid tumors. Jpn J Clin Oncol (2013) 43(11):1073–9. 10.1093/jjco/hyt124 24002900

[13] Porkholm M Valanne L Lonnqvist T Holm S Lannering B Riikonen P Radiation therapy and concurrent topotecan followed by maintenance triple anti-angiogenic therapy with thalidomide, etoposide, and celecoxib for pediatric diffuse intrinsic pontine glioma. Pediatr Blood Cancer (2014) 61(9):1603–9. 10.1002/pbc.25045 24692119

[14] Rizzo D Scalzone M Ruggiero A Maurizi P Attina G Mastrangelo S Temozolomide in the treatment of newly diagnosed diffuse brainstem glioma in children: a broken promise? J Chemother (2015) 27(2):106–10. 10.1179/1973947814Y.0000000228 25466729

[15] Roos DE Smith JG. Randomized trials on radioactive iodine ablation of thyroid remnants for thyroid carcinoma – a critique. Int J Radiat Oncol Biol Phys (1999) 44(3):493–5. 10.1016/S0360-3016(98)00553-7 10348276

[16] Veldhuijzen van Zanten SE Jansen MH Sanchez Aliaga E van Vuurden DG Vandertop WP Kaspers GJ. A twenty-year review of diagnosing and treating children with diffuse intrinsic pontine glioma in The Netherlands. Expert Rev Anticancer Ther (2015) 15(2):157–64. 10.1586/14737140.2015.974563 25435089

[17] Zaghloul MS Eldebawy E Ahmed S Mousa AG Amin A Refaat A Hypofractionated conformal radiotherapy for pediatric diffuse intrinsic pontine glioma (DIPG): a randomized controlled trial. Radiother Oncol (2014) 111(1):35–40. 10.1016/j.radonc.2014.01.013 24560760

[18] Zaky W Wellner M Brown RJ Bluml S Finlay JL Dhall G. Treatment of children with diffuse intrinsic pontine gliomas with chemoradiotherapy followed by a combination of temozolomide, irinotecan, and bevacizumab. Pediatr Hematol Oncol (2013) 30(7):623–32. 10.3109/08880018.2013.829895 24050762

[19] Freeman CR Perilongo G. Chemotherapy for brain stem gliomas. Childs Nerv Syst (1999) 15(10):545–53. 10.1007/s003810050542 10550585

[20] Maria BL Rehder K Eskin TA Hamed LM Fennell EB Quisling RG Brainstem glioma: I. Pathology, clinical features, and therapy. J Child Neurol (1993) 8(2):112–28. 10.1177/088307389300800203 8505473

[21] Donaldson SS Laningham F Fisher PG. Advances toward an understanding of brainstem gliomas. J Clin Oncol (2006) 24(8):1266–72. 10.1200/JCO.2005.04.6599 16525181

[22] Albright AL Price RA Guthkelch AN. Brain stem gliomas of children. A clinicopathological study. Cancer (1983) 52(12):2313–9. 10.1002/1097-0142(19831215)52:12<2313::AID-CNCR2820521226>3.0.CO;2-I 6640503

[23] Albright AL Packer RJ Zimmerman R Rorke LB Boyett J Hammond GD. Magnetic resonance scans should replace biopsies for the diagnosis of diffuse brain stem gliomas: a report from the children’s cancer group. Neurosurgery (1993) 33(6):1026–9. 10.1227/00006123-199312000-00010 8133987

[24] Schwartzentruber J Korshunov A Liu XY Jones DT Pfaff E Jacob K Driver mutations in histone H3.3 and chromatin remodelling genes in paediatric glioblastoma. Nature (2012) 482(7384):226–31. 10.1038/nature10833 22286061

[25] Wu G Broniscer A McEachron TA Lu C Paugh BS Becksfort J Somatic histone H3 alterations in pediatric diffuse intrinsic pontine gliomas and non-brainstem glioblastomas. Nat Genet (2012) 44(3):251–3. 10.1038/ng.1102 22286216 PMC3288377

[26] Cage TA Samagh SP Mueller S Nicolaides T Haas-Kogan D Prados M Feasibility, safety, and indications for surgical biopsy of intrinsic brainstem tumors in children. Childs Nerv Syst (2013) 29(8):1313–9. 10.1007/s00381-013-2101-0 23666401

[27] Grill J Puget S Andreiuolo F Philippe C MacConaill L Kieran MW. Critical oncogenic mutations in newly diagnosed pediatric diffuse intrinsic pontine glioma. Pediatr Blood Cancer (2012) 58(4):489–91. 10.1002/pbc.24060 22190243

[28] Kieran MW Time to rethink the unthinkable: upfront biopsy of children with newly diagnosed diffuse intrinsic pontine glioma (DIPG). Pediatr Blood Cancer (2015) 62(1):3–4. 10.1002/pbc.25266 25284709

[29] MacDonald TJ Diffuse intrinsic pontine glioma (DIPG): time to biopsy again? Pediatr Blood Cancer (2012) 58(4):487–8. 10.1002/pbc.24090 22331797

[30] Cheng Y Ng HK Zhang SF Ding M Pang JC Zheng J Genetic alterations in pediatric high-grade astrocytomas. Hum Pathol (1999) 30(11):1284–90. 10.1016/S0046-8177(99)90057-6 10571506

[31] Louis DN Rubio MP Correa KM Gusella JF von Deimling A. Molecular genetics of pediatric brain stem gliomas. Application of PCR techniques to small and archival brain tumor specimens. J Neuropathol Exp Neurol (1993) 52(5):507–15. 10.1097/00005072-199309000-00009 8103086

[32] Pollack IF Hamilton RL Finkelstein SD Campbell JW Martinez AJ Sherwin RN The relationship between TP53 mutations and overexpression of p53 and prognosis in malignant gliomas of childhood. Cancer Res (1997) 57(2):304–9. 9000573

[33] Sure U Ruedi D Tachibana O Yonekawa Y Ohgaki H Kleihues P Determination of p53 mutations, EGFR overexpression, and loss of p16 expression in pediatric glioblastomas. J Neuropathol Exp Neurol (1997) 56(7):782–9. 10.1097/00005072-199707000-00004 9210874

[34] Brennan CW Verhaak RG McKenna A Campos B Noushmehr H Salama SR The somatic genomic landscape of glioblastoma. Cell (2013) 155(2):462–77. 10.1016/j.cell.2013.09.034 24120142 PMC3910500

[35] Cancer Genome Atlas Research N. Comprehensive genomic characterization defines human glioblastoma genes and core pathways. Nature (2008) 455(7216):1061–8. 10.1038/nature07385 18772890 PMC2671642

[36] Verhaak RG Hoadley KA Purdom E Wang V Qi Y Wilkerson MD Integrated genomic analysis identifies clinically relevant subtypes of glioblastoma characterized by abnormalities in PDGFRA, IDH1, EGFR, and NF1. Cancer Cell (2010) 17(1):98–110. 10.1016/j.ccr.2009.12.020 20129251 PMC2818769

[37] Zarghooni M Bartels U Lee E Buczkowicz P Morrison A Huang A Whole-genome profiling of pediatric diffuse intrinsic pontine gliomas highlights platelet-derived growth factor receptor alpha and poly (ADP-ribose) polymerase as potential therapeutic targets. J Clin Oncol (2010) 28(8):1337–44. 10.1200/JCO.2009.25.5463 20142589

[38] Paugh BS Broniscer A Qu C Miller CP Zhang J Tatevossian RG Genome-wide analyses identify recurrent amplifications of receptor tyrosine kinases and cell-cycle regulatory genes in diffuse intrinsic pontine glioma. J Clin Oncol (2011) 29(30):3999–4006. 10.1200/JCO.2011.35.5677 21931021 PMC3209696

[39] Paugh BS Qu C Jones C Liu Z Adamowicz-Brice M Zhang J Integrated molecular genetic profiling of pediatric high-grade gliomas reveals key differences with the adult disease. J Clin Oncol (2010) 28(18):3061–8. 10.1200/JCO.2009.26.7252 20479398 PMC2903336

[40] Buczkowicz P Hoeman C Rakopoulos P Pajovic S Letourneau L Dzamba M Genomic analysis of diffuse intrinsic pontine gliomas identifies three molecular subgroups and recurrent activating ACVR1 mutations. Nat Genet (2014) 46(5):451–6. 10.1038/ng.2936 24705254 PMC3997489

[41] Taylor KR Mackay A Truffaux N Butterfield YS Morozova O Philippe C Recurrent activating ACVR1 mutations in diffuse intrinsic pontine glioma. Nat Genet (2014) 46(5):457–61. 10.1038/ng.2925 24705252 PMC4018681

[42] Wu G Diaz AK Paugh BS Rankin SL Ju B Li Y The genomic landscape of diffuse intrinsic pontine glioma and pediatric non-brainstem high-grade glioma. Nat Genet (2014) 46(5):444–50. 10.1038/ng.2938 24705251 PMC4056452

[43] Fontebasso AM Papillon-Cavanagh S Schwartzentruber J Nikbakht H Gerges N Fiset PO Recurrent somatic mutations in ACVR1 in pediatric midline high-grade astrocytoma. Nat Genet (2014) 46(5):462–6. 10.1038/ng.2950 24705250 PMC4282994

[44] Ederveen TH Mandemaker IK Logie C. The human histone H3 complement anno 2011. Biochim Biophys Acta (2011) 1809(10):577–86. 10.1016/j.bbagrm.2011.07.002 21782046

[45] Liang ML Ma J Ho M Solomon L Bouffet E Rutka JT Tyrosine kinase expression in pediatric high grade astrocytoma. J Neurooncol (2008) 87(3):247–53. 10.1007/s11060-007-9513-1 18193393

[46] Zhang L Chen LH Wan H Yang R Wang Z Feng J Exome sequencing identifies somatic gain-of-function PPM1D mutations in brainstem gliomas. Nat Genet (2014) 46(7):726–30. 10.1038/ng.2995 24880341 PMC4073211

[47] Paugh BS Zhu X Qu C Endersby R Diaz AK Zhang J Novel oncogenic PDGFRA mutations in pediatric high-grade gliomas. Cancer Res (2013) 73(20):6219–29. 10.1158/0008-5472.CAN-13-1491 23970477 PMC3800209

[48] Puget S Philippe C Bax DA Job B Varlet P Junier MP Mesenchymal transition and PDGFRA amplification/mutation are key distinct oncogenic events in pediatric diffuse intrinsic pontine gliomas. PLoS One (2012) 7(2):e30313. 10.1371/journal.pone.0030313 22389665 PMC3289615

[49] Pollack IF Finkelstein SD Woods J Burnham J Holmes EJ Hamilton RL Expression of p53 and prognosis in children with malignant gliomas. N Engl J Med (2002) 346(6):420–7. 10.1056/NEJMoa012224 11832530

[50] Aihara K Mukasa A Gotoh K Saito K Nagae G Tsuji S H3F3A K27M mutations in thalamic gliomas from young adult patients. Neuro Oncol (2014) 16(1):140–6. 10.1093/neuonc/not144 24285547 PMC3870821

[51] Bernstein BE Mikkelsen TS Xie X Kamal M Huebert DJ Cuff J A bivalent chromatin structure marks key developmental genes in embryonic stem cells. Cell (2006) 125(2):315–26. 10.1016/j.cell.2006.02.041 16630819

[52] Meissner A Mikkelsen TS Gu H Wernig M Hanna J Sivachenko A Genome-scale DNA methylation maps of pluripotent and differentiated cells. Nature (2008) 454(7205):766–70. 10.1038/nature07107 18600261 PMC2896277

[53] Reynolds N Salmon-Divon M Dvinge H Hynes-Allen A Balasooriya G Leaford D NuRD-mediated deacetylation of H3K27 facilitates recruitment of Polycomb Repressive Complex 2 to direct gene repression. EMBO J (2012) 31(3):593–605. 10.1038/emboj.2011.431 22139358 PMC3273378

[54] Sturm D Bender S Jones DT Lichter P Grill J Becher O Paediatric and adult glioblastoma: multiform (epi)genomic culprits emerge. Nat Rev Cancer (2014) 14(2):92–107. 10.1038/nrc3655 24457416 PMC4003223

[55] Bender S Tang Y Lindroth AM Hovestadt V Jones DT Kool M Reduced H3K27me3 and DNA hypomethylation are major drivers of gene expression in K27M mutant pediatric high-grade gliomas. Cancer Cell (2013) 24(5):660–72. 10.1016/j.ccr.2013.10.006 24183680

[56] Chan KM Fang D Gan H Hashizume R Yu C Schroeder M The histone H3.3K27M mutation in pediatric glioma reprograms H3K27 methylation and gene expression. Genes Dev (2013) 27(9):985–90. 10.1101/gad.217778.113 23603901 PMC3656328

[57] Venneti S Garimella MT Sullivan LM Martinez D Huse JT Heguy A Evaluation of histone 3 lysine 27 trimethylation (H3K27me3) and enhancer of Zest 2 (EZH2) in pediatric glial and glioneuronal tumors shows decreased H3K27me3 in H3F3A K27M mutant glioblastomas. Brain Pathol (2013) 23(5):558–64. 10.1111/bpa.12042 23414300 PMC3701028

[58] Venneti S Santi M Felicella MM Yarilin D Phillips JJ Sullivan LM A sensitive and specific histopathologic prognostic marker for H3F3A K27M mutant pediatric glioblastomas. Acta Neuropathol (2014) 128(5):743–53. 10.1007/s00401-014-1338-3 25200322 PMC4201755

[59] Lewis PW Muller MM Koletsky MS Cordero F Lin S Banaszynski LA Inhibition of PRC2 activity by a gain-of-function H3 mutation found in pediatric glioblastoma. Science (2013) 340(6134):857–61. 10.1126/science.1232245 23539183 PMC3951439

[60] Akbari MR Lepage P Rosen B McLaughlin J Risch H Minden M PPM1D mutations in circulating white blood cells and the risk for ovarian cancer. J Natl Cancer Inst (2014) 106(1):djt323 10.1093/jnci/djt323 24262437

[61] Richter M Dayaram T Gilmartin AG Ganji G Pemmasani SK Van Der Key H WIP1 phosphatase as a potential therapeutic target in neuroblastoma. PLoS One (2015) 10(2):e0115635. 10.1371/journal.pone.0115635 25658463 PMC4319922

[62] Zajkowicz A Butkiewicz D Drosik A Giglok M Suwinski R Rusin M. Truncating mutations of PPM1D are found in blood DNA samples of lung cancer patients. Br J Cancer (2015) 112:1114–20. 10.1038/bjc.2015.79 25742468 PMC4366904

[63] Kleiblova P Shaltiel IA Benada J Sevcik J Pechackova S Pohlreich P Gain-of-function mutations of PPM1D/Wip1 impair the p53-dependent G1 checkpoint. J Cell Biol (2013) 201(4):511–21. 10.1083/jcb.201210031 23649806 PMC3653305

[64] Kaplan FS Xu M Seemann P Connor JM Glaser DL Carroll L Classic and atypical fibrodysplasia ossificans progressiva (FOP) phenotypes are caused by mutations in the bone morphogenetic protein (BMP) type I receptor ACVR1. Hum Mutat (2009) 30(3):379–90. 10.1002/humu.20868 19085907 PMC2921861

[65] Petrie KA Lee WH Bullock AN Pointon JJ Smith R Russell RG Novel mutations in ACVR1 result in atypical features in two fibrodysplasia ossificans progressiva patients. PLoS One (2009) 4(3):e5005. 10.1371/journal.pone.0005005 19330033 PMC2658887

[66] Song GA Kim HJ Woo KM Baek JH Kim GS Choi JY Molecular consequences of the ACVR1(R206H) mutation of fibrodysplasia ossificans progressiva. J Biol Chem (2010) 285(29):22542–53. 10.1074/jbc.M109.094557 20463014 PMC2903413

[67] Kleihues P Ohgaki H. Primary and secondary glioblastomas: from concept to clinical diagnosis. Neuro Oncol (1999) 1(1):44–51. 10.1215/15228517-1-1-44 11550301 PMC1919466

[68] Rood BR MacDonald TJ. Pediatric high-grade glioma: molecular genetic clues for innovative therapeutic approaches. J Neurooncol (2005) 75(3):267–72. 10.1007/s11060-005-6749-5 16195804

[69] Hegi ME Diserens AC Gorlia T Hamou MF de Tribolet N Weller M MGMT gene silencing and benefit from temozolomide in glioblastoma. N Engl J Med (2005) 352(10):997–1003. 10.1056/NEJMoa043331 15758010

[70] Agnihotri S Burrell K Buczkowicz P Remke M Golbourn B Chornenkyy Y ATM regulates 3-methylpurine-DNA glycosylase and promotes therapeutic resistance to alkylating agents. Cancer Discov (2014) 4(10):1198–213. 10.1158/2159-8290.CD-14-0157 25100205 PMC4184920

[71] Yan H Parsons DW Jin G McLendon R Rasheed BA Yuan W IDH1 and IDH2 mutations in gliomas. N Engl J Med (2009) 360(8):765–73. 10.1056/NEJMoa0808710 19228619 PMC2820383

[72] Liu XY Gerges N Korshunov A Sabha N Khuong-Quang DA Fontebasso AM Frequent ATRX mutations and loss of expression in adult diffuse astrocytic tumors carrying IDH1/IDH2 and TP53 mutations. Acta Neuropathol (2012) 124(5):615–25. 10.1007/s00401-012-1031-3 22886134

[73] Sturm D Witt H Hovestadt V Khuong-Quang DA Jones DT Konermann C Hotspot mutations in H3F3A and IDH1 define distinct epigenetic and biological subgroups of glioblastoma. Cancer Cell (2012) 22(4):425–37. 10.1016/j.ccr.2012.08.024 23079654

[74] Harley CB Futcher AB Greider CW. Telomeres shorten during ageing of human fibroblasts. Nature (1990) 345(6274):458–60. 10.1038/345458a0 2342578

[75] Greider CW. Telomeres, telomerase and senescence. Bioessays (1990) 12(8):363–9. 10.1002/bies.950120803 2241933

[76] Kim NW Piatyszek MA Prowse KR Harley CB West MD Ho PL Specific association of human telomerase activity with immortal cells and cancer. Science (1994) 266(5193):2011–5. 10.1126/science.7605428 7605428

[77] Wright WE Piatyszek MA Rainey WE Byrd W Shay JW. Telomerase activity in human germline and embryonic tissues and cells. Dev Genet (1996) 18(2):173–9. 10.1002/(SICI)1520-6408(1996)18:2<173::AID-DVG10>3.0.CO;2-3 8934879

[78] Barszczyk M Buczkowicz P Castelo-Branco P Mack SC Ramaswamy V Mangerel J Telomerase inhibition abolishes the tumorigenicity of pediatric ependymoma tumor-initiating cells. Acta Neuropathol (2014) 128(6):863–77. 10.1007/s00401-014-1327-6 25120190 PMC4286630

[79] Mangerel J Price A Castelo-Branco P Brzezinski J Buczkowicz P Rakopoulos P Alternative lengthening of telomeres is enriched in, and impacts survival of TP53 mutant pediatric malignant brain tumors. Acta Neuropathol (2014) 128(6):853–62. 10.1007/s00401-014-1348-1 25315281

[80] Remke M Ramaswamy V Peacock J Shih DJ Koelsche C Northcott PA TERT promoter mutations are highly recurrent in SHH subgroup medulloblastoma. Acta Neuropathol (2013) 126(6):917–29. 10.1007/s00401-013-1198-2 24174164 PMC3830749

[81] Caretti V Bugiani M Freret M Schellen P Jansen M van Vuurden D Subventricular spread of diffuse intrinsic pontine glioma. Acta Neuropathol (2014) 128(4):605–7. 10.1007/s00401-014-1307-x 24929912 PMC4161623

[82] Sethi R Allen J Donahue B Karajannis M Gardner S Wisoff J Prospective neuraxis MRI surveillance reveals a high risk of leptomeningeal dissemination in diffuse intrinsic pontine glioma. J Neurooncol (2011) 102(1):121–7. 10.1007/s11060-010-0301-y 20623246

[83] Yoshimura J Onda K Tanaka R Takahashi H. Clinicopathological study of diffuse type brainstem gliomas: analysis of 40 autopsy cases. Neurol Med Chir (2003) 43(8):375–82. 10.2176/nmc.43.375 12968803

[84] Sufit A Donson AM Birks DK Knipstein JA Fenton LZ Jedlicka P Diffuse intrinsic pontine tumors: a study of primitive neuroectodermal tumors versus the more common diffuse intrinsic pontine gliomas. J Neurosurg Pediatr (2012) 10(2):81–8. 10.3171/2012.3.PEDS11316 22747092 PMC4690743

[85] Saratsis AM Kambhampati M Snyder K Yadavilli S Devaney JM Harmon B Comparative multidimensional molecular analyses of pediatric diffuse intrinsic pontine glioma reveals distinct molecular subtypes. Acta Neuropathol (2014) 127(6):881–95. 10.1007/s00401-013-1218-2 24297113 PMC4028366

